# Contamination by Sewage

**Published:** 1888-06

**Authors:** 


### CONTAMINATION BY SEWAGE.

The Illinois State Board of Health has begun an investigation in order to determine how far down the Illinois River the water is contaminated by the sewage which is emptied into it at Chicago.

Every one knows that sooner or later the organic matter in contaminated streams is oxidized and removed and the water becomes as pure as before. But how much exposure to the air is required and how long it takes is a matter upon which there is still great difference of opinion among hygienists.

Many of our readers may know that about three years ago a preliminary study was made by Professor J. H. Long in this direction. Many valuable facts were learned from this study, but much was left untouched; for instance, nothing was done to ascertain how much contamination was received from the towns on the banks, or what was the influence of the various tributaries, whether to increase the contamination or to dilute it.

All these subjects will be studied at this time.

In addition to the chemical investigation by Professor Long a biological study will be made by Dr. Lester Curtis.

A part, at least, of the injurious effects of sewage comes from the process of decomposition.

Sewage which has become thoroughly decomposed is harmless; fresh dishwater and slops are also harmless. The same thing can be said of urine and even of nightsoil. It is only while undergoing decomposition that these substances are poisonous, and this decomposition is always accompanied by germs of putrefaction. An attempt will be made by Dr. Curtis to ascertain the number of germs in the various samples of water, and to ascertain as far as possible their nature. These will serve as an index of the unwholesomeness of the water.

The process used is "plate cultures" on neutral peptonized gelatine. The water, when bad, is diluted with boiled distilled water and a measured quantity is added to the gelatine and then spread over the plates. These are then placed in a culture-chamber and examined from time to time. Every precaution is, of course, taken to avoid mixture with other germs by sterilizing the diluting water, the gelatine, the plates, and other apparatus used.

It is hoped that the whole investigation will lead to facts of practical value as well as of scientific interest. The board is to be commended for its enterprise and public spirit in starting the work.

